# High percentages and activity of synovial fluid NK cells present in patients with advanced stage active Rheumatoid Arthritis

**DOI:** 10.1038/s41598-018-37448-z

**Published:** 2019-02-04

**Authors:** Rachel Yamin, Orit Berhani, Hagit Peleg, Suhail Aamar, Natan Stein, Moriya Gamliel, Issam Hindi, Anat Scheiman-Elazary, Chamutal Gur

**Affiliations:** 1 The Lautenberg Center for General and Tumor Immunology, The BioMedical Research Institute Israel-Canada of the Faculty of Medicine (IMRIC), The Hebrew University-Hadassah Medical School, Jerusalem, 91120 Israel; 2 The Internal medicine department and the Rheumatology unit, Hadassah Medical Center, Jerusalem, 91120 Israel

## Abstract

Rheumatoid Arthritis (RA) causes chronic inflammation of joints. The cytokines TNFα and IFNγ are central players in RA, however their source has not been fully elucidated. Natural Killer (NK) cells are best known for their role in elimination of viral-infected and transformed cells, and they secrete pro-inflammatory cytokines. NK cells are present in the synovial fluids (SFs) of RA patients and are considered to be important in bone destruction. However, the phenotype and function of NK cells in the SFs of patients with erosive deformative RA (DRA) versus non-deformative RA (NDRA) is poorly characterized. Here we characterize the NK cell populations present in the blood and SFs of DRA and NDRA patients. We demonstrate that a distinct population of activated synovial fluid NK (sfNK) cells constitutes a large proportion of immune cells found in the SFs of DRA patients. We discovered that although sfNK cells in both DRA and NDRA patients have similar phenotypes, they function differently. The DRA sfNK secrete more TNFα and IFNγ upon exposure to IL-2 and IL-15. Consequently, we suggest that sfNK cells may be a marker for more severely destructive RA disease.

## Introduction

Rheumatoid arthritis (RA) is a chronic autoimmune disease that affects ~1% of the adult population. The synovium is the primary site of the inflammatory process, and synovitis can lead to erosion of the joint surface causing deformity and loss of function. Approximately 40% of patients with this disease become disabled after ten years^1^. Despite advances in our understanding of the pathogenesis of RA, the cause of the disease is still unknown. It is hypothesized, however, that both genetic and environmental factors are required for disease development. Immune system abnormalities also contribute to disease propagation, and multiple arms of the immune system have been shown to participate in the autoimmune process of RA. These include T and B cells, antigen-presenting cells and various cytokines^2^. Growing evidence exposes the importance of Natural Killer (NK) cells, lymphocytes of the innate immune system, in autoimmune diseases^3^. NK cells were originally characterized for their capacity to kill transformed and virus-infected cells^4–6^. They distinguish abnormal cells from healthy cells by balancing signals received from inhibitory and activating receptors found on their surface^4–8^. NK cells in the peripheral blood are divided into two major subsets, based on the density and expression of the surface molecules CD56 and CD16 (FcγRIIIA): CD56^dim^, which express high levels of CD16 (CD56^dim^CD16^+^); and CD56^bright^, which are negative for or express low levels of CD16 (CD56^bright^CD16^−/dim^)^9,10^. NK cell cytolytic activity is mostly confined to the blood CD56^dim^ subset, whereas cytokine production is generally assigned to CD56^bright^ cells^9^. The two NK cell subsets differentially express various chemokine receptors which attract them to various organs. Thus, the CD56^dim^ population is abundant in the blood (~90%), while the CD56^bright^ population resides in secondary lymph nodes, in sites of peripheral inflammation, and in the decidua during pregnancy^10–13^. NK cells also have important regulatory functions mediated by the secretion of cytokines, such as IFNγ and TNFα^5^. In addition, although NK cells are regarded as innate immune cells, recent findings have demonstrated that NK cells display ‘adaptive’ features and can mount memory responses following specific activation by chemical haptens, viruses, or even nonspecific activation by cytokines^14,15^.

Several reports have shown enrichment of NK cells within inflamed joints of patients with various arthritic diseases, including RA patients^16–18^. It was also shown that synovial fluid NK (sfNK) cells co-cultured with monocytes *in vitro* could trigger their differentiation into osteoclasts^19^. Furthermore, in a mouse model of arthritis, depletion of NK cells from mice before the induction of arthritis almost completely prevented bone erosions^19^.

Dalbeth *et al*.^17^ described the phenotype of NK cells derived from patients with inflammatory arthritis including 15 patients with RA. They found that in the SFs of those patients there was significant expansion of NK cells that expressed high levels of CD56 and low levels of CD16. They further showed that the majority of these cells express the chemokine receptors CCR5 and CXCR3, whereas a small percentage of the sfNK cells expressed the killer Ig–like receptors. Using intracellular FACS staining of paired samples of peripheral blood and sfNK cells derived from a patient with RA, they showed an increase in IFNγ expression following incubation with IL-12 and IL-15. Unfortunately, the above study did not provide sufficient or  specific information regarding the RA patients analyzed, thus the levels of deformations, erosions, disease activity or severity, and titers of autoantibodies (RF and anti-CCP) were missing^17^. Furthermore, only 60% of the patients were seropositive for RF, most of the RA patients in the study were on disease-modifying antirheumatic drugs (DMARDs) monotherapy, and none of the patients were treated with biological treatments, suggesting mild RA disease.

Since sfNK may play an important role in destruction of joints, observed in both *in vitro* and *in vivo* models of arthritis, our aim was to characterize the phenotype and function of blood and sfNK cells of RA patients in correlation with disease severity. In this study we analyzed the blood and sfNK cells of RA patients with advanced deformative (deformations which were classical for RA) and erosive (radiographic evidence of bony erosion, which is the hallmark of severe RA) disease (DRA), and in patients with non deformative disease (NDRA). We show that the sfNK cell subset is unlike any population documented in any other organ and is enriched in patients with DRA. We demonstrate that although sfNK cells in DRA and NDRA patients have similar receptor expression and activation markers, the ability of sfNK cells in DRA patients to secrete TNFα and IFNγ upon exposure to IL-2 and IL-15 is higher.

By understanding the behavior of sfNK cells and their contribution to the progression of the disease we may have the potential to influence the course of treatment for severely ill patients with DRA.

## Results

### Increase of NK cell percentages in the peripheral blood and SFs of DRA patients

**Figure 1:**
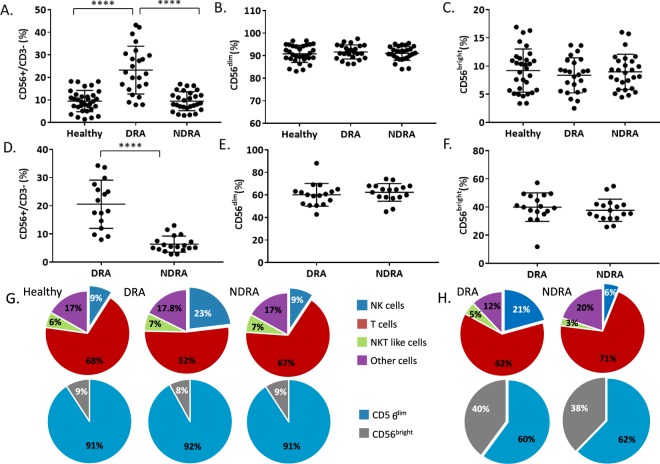
Increase of NK cell percentages in the peripheral blood and SFs of DRA patients. (**A–C**) Peripheral blood mononuclear cells (PBMCs) were isolated from healthy controls (n = 32), DRA patients (n = 24), and NDRA patients (n = 27). The mean percentages and standard deviations of NK cells (CD56^+^/CD3^−^, **A**) and different NK cell sub-populations (CD56^dim^, **B**, CD56^bright^, **C**) in the blood of each group are shown in the graphs. *The ratio of CD56^+^/CD3^−^ varies significantly among the three groups (****p < 10^−11^). Two of the three post hoc pairwise tests (blood DRA versus healthy or blood DRA versus NDRA) were also very highly significant (****p < 10^−5^). (**D**–**F**) Mononuclear cells were isolated from SFs of DRA patients (left panels, n = 16), and NDRA patients (right panels, n = 18) described in (**A**–**C**). The mean percentages and standard deviations of sfNK cells (CD56^+^/CD3^−^, **D**) and different NK cell sub-populations (CD56^dim^, **E**, CD56^bright^, **F**) in the SFs of each group are shown in the graphs.****p < 10^−6^ (**G**) Pie charts summarizing the different sub-populations of immune cells derived from the blood of healthy controls, DRA and NDRA patients (upper charts), and the different sub-populations of blood CD56^dim^ and CD56^bright^ NK cells (lower charts) derived from healthy controls, DRA and NDRA patients described in (**A–C)**. (**H**) Pie charts summarizing the different sub-populations of immune cells derived from the SFs of DRA and NDRA patients (upper charts), and the different sub-populations of SFs CD56^dim^ and CD56^bright^ NK cells (lower charts) derived from DRA and NDRA patients described in (**D**–**F**).

To analyze whether NK cells may be involved in the severity of RA, we initially assessed the levels of NK cells and the distribution of NK cell subsets in the blood of healthy controls, DRA and NDRA patients. The DRA, NDRA, and healthy control patients reported in this study are described in Methods and in Tables 1–3, respectively. As can be seen in Fig. 1A, the percentage of NK cells (described as CD56^+^/CD3^−^) was doubled in the peripheral blood of DRA patients as compared to healthy controls and NDRA patients. However, the distribution of the two NK cell subsets ~90% CD56^dim^CD16^+^ (CD56^dim^) and ~10% CD56^bright^CD16^−/dim^ (CD56^bright^) remained unchanged between the patient groups (Fig. 1B,C, respectively). Next, we characterized the NK cell percentages in the SFs of DRA and NDRA patients. Similarly to the results seen in the blood, three times as many NK cells were found in the SFs of DRA patients as compared to NDRA patients (Fig. 1D). The higher percentages of NK cells in both the blood and SFs of DRA patients when compared with NDRA patients hints at the importance of NK cells in the pathogenesis of joint destruction. Interestingly, the distribution of the two NK cells subsets in the SFs of both DRA and NDRA patients changed dramatically when compared with the peripheral blood NK cells. In the SFs, the distribution was about 60/40 between CD56^dim^ and CD56^bright^ in DRA and NDRA patients (Fig. 1E,F, respectively). A summary of the results is presented in Fig. 1G,H.

**Table 1:** The DRA patients enrolled in the study.

No.	Age/Sex	Disease duration	RF	ACPA	DAS28CRP	DMARDs	Biological treatments	Prednisone
#1	84/M	26	Negative	Negative	6.8	HCQ, LEF, MTX, Gold	Anti TNF, ABT	7.5 mg/d
#2	66/F	10	155	>160	5.1	HCQ, MTX		No
#3	57/M	15	185	>130	7.53	HCQ, LEF, MTX, SAL, AZA, CPM, CYC	Anti TNF, RIT, TOC, ABT, INN	10 mg/d
#4	60/F	32	30	118	5.25	HCQ, LEF, MTX, SAL,	Anti TNF, RIT	10 mg/d
#5	61/F	8	281	ND	5.8	HCQ, MTX		10 mg/d
#6	68/F	7.5	193	114	5.79	MTX, SAL		15 mg/d
#7	69/F	20	284	Negative	4.9	HCQ, MTX, SAL	Anti TNF, RIT	5 mg/d
#8	54/M	7	83	225	5.1	HCQ, MTX, SAL	Anti TNF, ABT	5 mg/d
#9	62/F	5	40	25	5.18	HCQ, MTX, AZA	Anti TNF	No
#10	60/M	16	455	>170	6.85	MTX, LEF		15 mg/d
#11	62/F	25	40	28	4.1	HCQ, MTX	Anti TNF	5 mg/d
#12	83/F	8	87	36.8	4.77	HCQ, MTX, SAL	Anti TNF, RIT, TOC, ABT	5 mg/d
#13	52/F	20	288	>160	3.9	HCQ, MTX, SAL	Anti TNF, RIT	10 mg/d
#14	43/F	10	45	>160	4.4	HCQ, LEF, SAL		5 mg/d
#15	79/M	20	280	ND	4.42	MTX		5 mg/d
#16	66/M	17	32	>250	4.35	HCQ, MTX, SAL, LEF		5 mg/d
#17	64/F	30	247	>140	6.37	HCQ, MTX	Anti TNF, RIT, TOC	20 mg/d
#18	34/M	4	220	>160	5.2	HCQ, MTX, LEF	Anti TNF	No
#19	76/F	36	65	ND	5.5	HCQ, MTX		5 mg/d
#20	66/F	15	Negative	Negative	3.32	HCQ, MTX, SAL	Anti TNF	No
#21	67/M	13	89	>140	3.8	HCQ, MTX, SAL, LEF	Anti TNF	5 mg/d
#22	72/M	3	35	>160	5.47	HCQ, MTX, LEF	RIT, ABT	7.5 mg/d
#23	71/M	13	78	>250	2.4	HCQ, MTX, SAL, LEF		No
#24	73/F	21	339	>250	4.6	HCQ, MTX, LEF	Anti TNF	5 mg/d

RF = Rheumatoid factor; ACPA = Anti-citrullinated protein antibodies; DAS28 CRP = Disease Activity Score in 28 joints using the C-reactive protein level; ND = Not determined; DMARD = Disease-modifying anti-rheumatic drugs: HCQ = Hydroxychloroquine; LEF = Leflunomide (Arava); MTX = Methotrexate;CPM = Cyclophosphamide; SAL = Sulfasalazine; AZA = Azathioprine (Imuran); CYC = Cyclosporine; TNF = Tumor necrosis factor; ABT = Abatacept (Orencia); RIT = Rituximab (Rituxan); TOC = Tocilizumab; INN = Tofacitinib (Xeljanz).

**Table 2:** The NDRA patients enrolled in this study.

No.	Age/Sex	Disease duration	RF	ACPA	DAS28CRP	DMARDs	Biological treatments	Prednisone
#1	58/F	7	220	65	4.9	HCQ, LEF, MTX	Anti TNF	5 mg/d
#2	54/F	15	22.5	>150	3.3	HCQ, MTX, SAL	Anti TNF, TOC	No
#3	69/F	17	110	108	4.4	HCQ, MTX, SAL	Anti TNF, TOC	10 mg/d
#4	57/M	5	63	205	3.1	HCQ, MTX, SAL		10 mg/d
#5	66/F	9	426	127	5.13	HCQ, MTX, SAL	Anti TNF, TOC, ABT	No
#6	46/F	15	39	41.6	5.8	HCQ, MTX, SAL	Anti TNF, ABT	5 mg/d
#7	68/F	12	48	ND	3.81	HCQ, MTX, LEF		5 mg/d
#8	48/F	6	Negative	Negative	4.2	HCQ, MTX, SAL		5 mg/d
#9	75/M	11	55	ND	5.9	HCQ, MTX		5 mg/d
#10	78/M	12	Negative	Negative	5.4	HCQ, MTX, SAL	Anti TNF	No
#11	54/F	9	57	>200	6.09	HCQ, MTX, SAL, LEF	Anti TNF	7.5 mg/d
#12	55/F	20	337	83.5	5.64	HCQ, MTX, SAL, LEF	Anti TNF, RIT, TOC, ABT, INN	10 mg/d
#13	48/F	10	75	>160	4.34	HCQ, MTX, LEF	Anti TNF	10 mg/d
#14	51/F	7	Negative	Negative	4.6	HCQ, MTX, SAL	Anti TNF	5 mg/d
#15	33/F	11	85	>200	5.3	HCQ, MTX, SAL	Anti TNF, TOC	10 mg/d
#16	57/F	4	150	162	6.2	HCQ, MTX		No
#17	65/F	21	Negative	>150	4.4	HCQ, MTX	Anti TNF	No
#18	56/M	8	250	>200	5.04	HCQ, MTX, LEF	Anti TNF, TOC	10 mg/d
#19	58/F	16	328	>150	3.4	HCQ, MTX		5 mg/d
#20	65/F	15	89	NA	3.32	HCQ, MTX, AZA	Anti TNF, ABT	No
#21	22/M	3	Negative	Negative	3.1	HCQ		No
#22	67/M	3	28	>160	4.86	HCQ, MTX		5 mg/d
#23	59/F	9	114	46	4.47	HCQ, MTX, LEF	Anti TNF, ABT, TOC, INN	2.5 mg/d
#24	86/M	5	43	ND	2.35	HCQ, MTX		No
#25	54/F	20	94	ND	1.9	HCQ, MTX, LEF		5 mg/d
#26	38/F	3	153	ND	4.07	HCQ, MTX, SAL	Anti TNF	10 mg/d
#27	67/M	30	250	>150	5.9	HCQ, MTX	RIT	10 mg/d

RF = Rheumatoid factor; ACPA = Anti-citrullinated protein antibodies; DAS28 CRP = Disease Activity Score in 28 joints using the C-reactive protein level; ND = Not determined; DMARD = Disease-modifying anti-rheumatic drugs: HCQ = Hydroxychloroquine; LEF = Leflunomide (Arava); MTX = Methotrexate; CPM = Cyclophosphamide; SAL = Sulfasalazine; AZA =  Azathioprine (Imuran); CYC = Cyclosporine; TNF = Tumor necrosis factor; ABT = Abatacept (Orencia); RIT = Rituximab (Rituxan); TOC = Tocilizumab; INN = Tofacitinib (Xeljanz).

**Table 3:** Controls enrolled in this study.

No.	Age/Sex	No.	Age/Sex
#1	35/F	#17	48/F
#2	51/F	#18	72/F
#3	37/M	#19	64/M
#4	52/F	#20	61/F
#5	42/M	#21	65/F
#6	67/F	#22	51/F
#7	28/M	#23	37/M
#8	59/F	#24	52/F
#9	56/M	#25	75/F
#10	65/F	#26	67/F
#11	57/F	#27	68/M
#12	61/F	#28	59/F
#13	79/M	#29	56/M
#14	62/F	#30	32/F
#15	75/M	#31	57/F
#16	68/F	#32	61/F

### NK cells in the synovial fluids of both DRA and NDRA patients are activated

**Figure 2:**
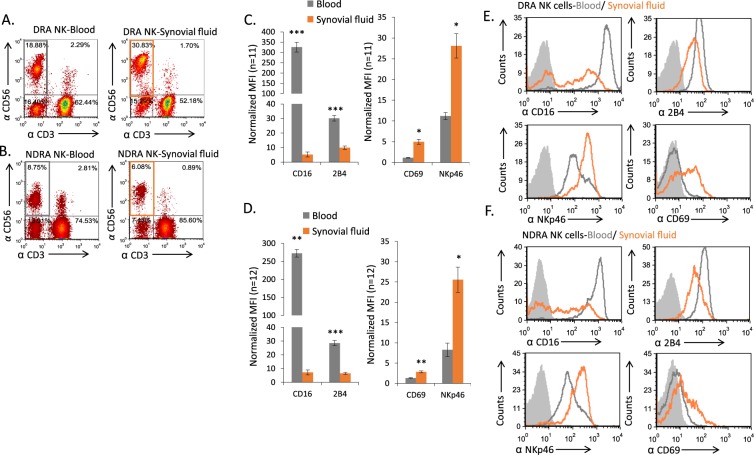
Alterations in NK receptors expressed on cells in blood and SFs of DRA and NDRA patients. (**A,B**) NK cells from peripheral blood (A, left, gray rectangle) or SFs (A, right, orange rectangle) of DRA, and blood and SFs NK cells of NDRA patients (B, left for blood, right for SFs) were double stained with anti CD56, anti CD3, and then triple stained with various monoclonal antibodies directed against different NK receptors. (**C**) Summary of data from FACS stainings of 11 DRA patients for the CD16, 2B4, NKp46 receptors and CD69 are presented as mean ± SE. (**D**) Summary of data from FACS stainings of 12 NDRA patients for the CD16, 2B4, NKp46 receptors and CD69 are presented as mean ± SE. Normalized MFI represents the specific MFI of an individual staining divided by the MFI of the background staining with an isotype control. Representative FACS staining of a DRA and NDRA patient is shown in E, and F, respectively. Open gray histograms show the staining of the receptors on cells from the peripheral blood. Open orange histograms indicate cells from the SFs. Filled gray histograms show the background staining of cells from the SFs with an isotype control. The backgrounds of the PBMCs were similar to the SF cells and are not shown in the figure. *p < 0.05. **p < 0.005. ***p < 0.0005.

Our next step was to analyze the NK cells’ receptor repertoire in the various patient groups. For this we isolated NK cells from the blood of healthy controls (data not shown), DRA, and NDRA patients (Fig. 2A,B left panels, respectively), and NK cells from SFs of DRA and NDRA patients (Fig. 2A,B right panels, respectively). Similar to the results in Fig. 1, more NK cells are present in the blood and SFs of patients with DRA as compared to NDRA patients (Fig. 2A versus 2B). Furthermore, in line with Fig. 1, in both DRA and NDRA patients, the NK cells in the blood were mainly from the CD56^dim^ population (around 90%), whereas, in the SFs of the inflamed joints the CD56^bright^ subset of NK cells was greatly expanded (Fig. 2A,B, left versus right panels).

Next, we proceeded to stain the NK cells from the blood and SFs of DRA and NDRA patients for a large repertoire of activating and inhibitory NK cell receptors. In both DRA and NDRA, only four receptors had significant differential expression between the blood and SFs, whereas the rest remained unchanged (Fig. S1A for DRA and S1B for NDRA patients). There were significant decreases in the expression levels of CD16 and 2B4, and conversely, significant increases in CD69 and NKp46 expression on NK cells from the SFs compared to those in the blood (Fig. 2C DRA,Fig. 2D NDRA). Representative FACS stainings of these observations from a DRA patient and an NDRA patient are presented in Fig. 2E,F, respectively.

We also compared the expression levels of the above receptors on blood NK cells from healthy controls, DRA and NDRA patients, and saw that only CD16 was differentially expressed (Fig. S1C). Furthermore, we observed two trends: a decrease in DNAM-1 expression and an increase in NKG2D expression on sfNK cells compared to blood NK; however, the changes were not statistically significant in both patient groups (Fig. S1). Lastly, we checked the expression levels of two receptors, NKG2A and NKp44, which were previously shown to be significantly increased on sfNK cells when compared to blood NK cells^17,20^. In both DRA and NDRA patients, we did not observe changes in the expression levels of these receptors (Fig. S1A,B, respectively).

Taken together, since CD69 and NKp46, which are markers of activation^21,22^, were highly elevated, we concluded that the NK cell populations in the SFs are in an activated state.

### The phenotypes of synovial fluid NK cells derived from DRA and NDRA patients are distinct but similar

**Figure 3:**
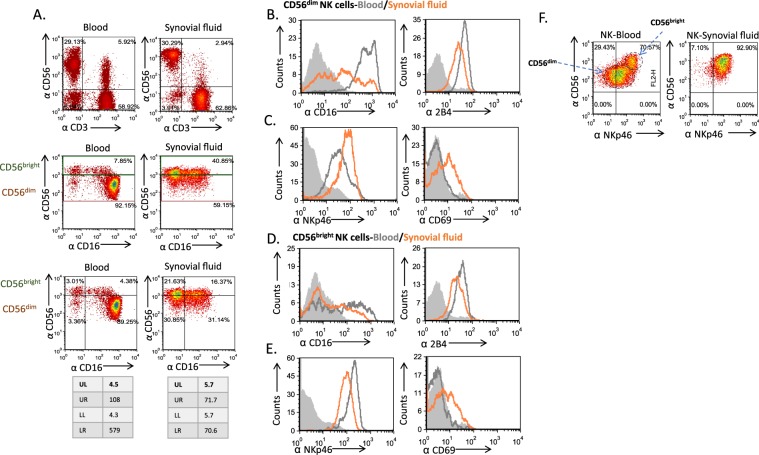
Blood and sfNK cell subsets are different. (**A–C**) Difference and similarity between subsets of blood and sfNK cells (**A**) Representative staining of mononuclear cells isolated from peripheral blood (left panels) and SFs (right panels) of DRA patient with anti CD56, anti CD3 and anti CD16. Following CD56^+^/CD3^−^ (NK cells) gating (upper panels), the CD56^dim^ and CD56^bright^ NK cells were gated (middle panels). The percentages and the MFI of the CD16 expression on the NK cell subsets are shown in the lower panels. Analysis of stainings of NK cell subsets of the study patients is shown in Fig. 1 (**B**) Mononuclear cells from peripheral blood or SFs of DRA patients were quadruple stained with anti CD56, anti CD3, anti CD16 and various monoclonal antibodies directed against the different NK receptors. CD56^dim^ and CD56^bright^ NK cells were gated, and the expression of NK receptors CD16 (**B**,**D**, left), 2B4 (**B**,**D**, right), NKp46 (**C**,**E**, left), and CD69 (**C**,**E**, right) was determined. Figure shows staining of cells from one representative donor out of 7 described in Fig. 2 that were tested. Open gray histograms and open orange histograms show the staining of the receptors on cells from the peripheral blood and the SFs, respectively. Filled gray histograms show the background staining of cells from the SFs with an isotype control. The backgrounds of the PBMCs were similar to the SFs cells and are not shown in the figure. (**F**) Representative triple staining with anti CD56, anti CD3, and NKp46 on blood and sfNK cells derived from a DRA patient. The two NK cell subsets are marked with circles. Figure shows staining of cells from one representative donor out of 7 described in Fig. 2 that were tested.

To further characterize the NK cell population residing within the SFs, we next repeated the FACS stainings performed in Fig. 2 but with a focus on the NK cell subsets. As we demonstrated in Fig. 2A,B, sfNK cells are significantly enriched with the CD56^bright^ population (Fig. 3A, upper panels, representative staining from a DRA patient), and the expression of CD16 (which is used for a clearer separation of the NK subsets^10^, Fig. 3A, middle panels) is differentially expressed between blood and sfNK cells (Fig. 3A, lower panels). Of blood CD56^bright^ cells about 50% expressed no or low levels of CD16, whereas above 95% of the blood CD56^dim^ cells have high expression of CD16. In contrast, both sfNK cell subsets expressed no or low levels of CD16. Out of the 3 other receptors with differential expression on NK cells found in blood and SFs (Fig. 2), only NKp46 expression varied between the CD56^dim^ and CD56^bright^ populations in both DRA and NDRA patients (Fig. 3B–E for DRA, Fig. S2A–D for NDRA). In the CD56^dim^ population, NKp46 expression is increased on NK cells from the SFs when compared to the blood NK cells (Fig. 3C,F for DRA, S2B for NDRA). In contrast, in the CD56^bright^ population, NKp46 expression is higher on NK cells from the blood when compared to the sfNK cells (Fig. 3E,F for DRA, S2D for NDRA). Nevertheless, the overall expression of NKp46 is significantly increased on NK cells found in the SFs of both DRA and NDRA patients (Fig. 2C,D).

NK cells migrate from the blood to inflamed areas. As such, we wanted to investigate whether the NK cells residing within the SFs of DRA and NDRA patients express a distinct chemokine receptor profile. Additionally, we analyzed the expression of NK cell receptors involved in NK cell function.

**Figure 4:**
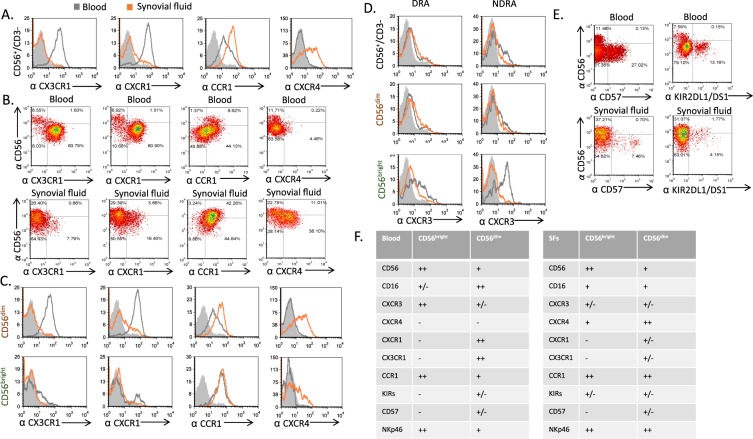
Alterations in NK receptors expressed on CD56^dim^ and CD56^bright^ subsets present in the blood and SFs of DRA and NDRA patients. (**A–C**) Representative FACS analysis of the expression level of various chemokine receptors on whole NK cells (**A,B**), CD56^dim^ (C, upper panels) and CD56^bright^ (C, lower panels) NK subsets from the blood (open gray histograms) or the SFs (open orange histograms) of DRA patient (staining of representative NDRA patient is shown in Fig. S3A). Filled gray histograms show the background staining of cells from the SFs with an isotype control. Figure shows staining of cells from one representative donor out of 6 that were tested. (**D**) Representative FACS analysis of the expression level of CXCR3 chemokine receptor on whole NK cells (histograms, upper panels), CD56^dim^ (middle panels) and CD56^bright^ (lower panels) NK subsets from the blood (open gray histograms) or the SFs (open orange histograms) of DRA and NDRA patients. Filled gray histograms show the background staining of cells from the SFs with an isotype control. Figure shows staining of cells from one representative donor out of 6 that were tested. (**E**) FACS staining of blood (upper panels) and sfNK cells (lower panels) of DRA patient with anti-CD3, anti-CD56 and specific antibodies against CD57 (left panels), and various KIRs receptors (right panels), with KIR2DL1/DS1 shown as a representative. A gate was set on CD56^+^/CD3^−^ (NK cells), versus CD57 or specific KIR is shown. Figure shows staining of cells from one representative donor (for each receptor) out of 6 that were tested. Similar staining of blood and sfNK cells of an NDRA patient is shown in Supplementary Fig. 3C. (**E**) Table summarizing differences between blood (left) and SFs (right) CD56^bright^ and CD56^dim^ NK cells ((++, strong expression (bright); +, weak expression (dim); +/−, expression only on a subpopulation; −, no expression)).

To begin our analysis, we stained blood and sfNK cells from DRA and NDRA patients against a range of chemokine receptors, where only four were observed to significantly change their levels of expression. In both patient populations there were decreases in CX3CR1 and CXCR1, and an increase in CCR1 and CXCR4 when comparing sfNK cells to blood NK cells (Fig. 4A,B for DRA, and S3A, upper panel for NDRA). These chemokine receptors are generally expressed differentially on blood NK cells subsets. Previous studies have shown that blood CD56^dim^ NK cells express high levels of CX3CR1, CXCR1, and intermediate levels of CXCR3 and CXCR2 receptors^9,23^. In contrast, blood CD56^bright^ NK cells express low amounts of CX3CR1 or CXCR1, but express higher levels of CCR1 and CXCR3^9^. We initially confirmed previous observations and saw that the blood CD56^dim^ subpopulation all express CX3CR1 and CXCR1 in both DRA and NDRA patents (Fig. 4B,C for DRA, S3A, middle panel for NDRA). Intermediate levels of CXCR2 were also observed on the blood CD56^dim^ subset (Fig. S3B, upper panel, representative staining from a DRA patient). As expected, there were intermediate levels of CCR1 and CXCR3 expression on the blood CD56^dim^ subpopulation, but high levels of both on the blood CD56^bright^ subpopulations (Fig. 4B–D for DRA, 4D, and Fig. S3A for NDRA).

When we analyzed the chemokine receptor profile of the sfNK cells in both DRA and NDRA patients, we initially saw that the CD56^dim^ subpopulation expresses low levels of CX3CR1 and CXCR1 and that the CD56^bright^ NK cells have almost no expression of these receptors (Fig. 4B,C for DRA, Fig. 4D, and S3A for NDRA). Interestingly, in both patient groups there was a significant decrease in CXCR3 expression on the CD56^bright^ sfNK cells, compared to blood CD56^bright^ population (Fig. 4D), whereas the CCR1 expression on sfNK cells remained high on both NK cell subsets (and increased relatively to the blood on the CD56^dim^ sfNK cells). We further observed upregulation of CXCR4 on the majority of sfNK cell population, and of CCR5 and CCR7 on the minority of sfNK cell population, compared with the blood NK cells in both DRA and NDRA patients (Figs 4A–C, S3A,B).

Other markers that can differentiate between blood CD56^dim^ and CD56^bright^ NK cells are CD57, a marker of highly mature NK cells^24^, and the KIRs, which are absent from CD56^bright^ NK cells, but are found on various proportions of CD56^dim^ cells^24,25^. We found that CD57 was expressed exlusively on CD56^dim^ NK cells in the blood (Fig. 4E for DRA, S3C for NDRA), but in the SFs the expression was low (Figs 4E, S3C). KIR expression was mainly restriced (as previously reported)^25^ to the CD56^dim^ NK population in the blood, yet in the SFs, both populations showed little KIR expression (Fig. 4E for DRA, S3C for NDRA).

A summary of receptors that are differentialy expressed on CD56^dim^ and CD56^bright^ blood and sfNK cells of both DRA and NDRA patients is shown in Fig. 4F (left and right, respectively).

### sfNK cells in DRA and NDRA patients are functionally distinct

**Figure 5:**
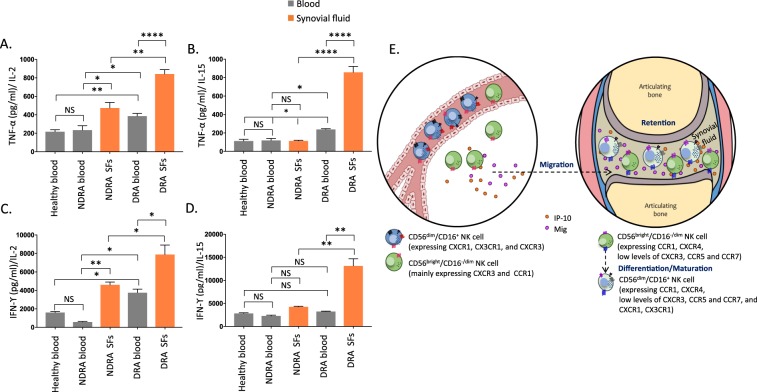
sfNK cells from DRA patients secrete higher levels of TNFα and IFNγ compared with sfNK cells from NDRA patients following incubation with IL-2 or IL-15. (**A**) NK cells sorted from a pool of blood of 6 normal controls and a pool of blood and SFs of 9 DRA and 10 NDRA patients described in Tables 1–3, incubated with either rhIL-2 or rhIL-15 following 48 h of standardization (dividing equal number of cells per well from each condition before starting the ELISA experiments). TNF-α (**A,B**) and IFN-γ (**C,D**) secretion in supernatants of NK cells isolated from blood of normal controls, DRA or NDRA patients, or from SFs of DRA and NDRA patients, incubated with either IL-2 (**A,C**), or IL-15 (**B,D**) was detected by ELISA and represented as pg/ml. Figure shows one representative experiment out of 4 performed. The TNF-α and IFN-γ dosage varies significantly among the groups (*p < 10^−8^) as determined by a One-Way ANOVA test. Various post hoc pairwise tests were highly significant after correcting for multiple comparisons. *p < 0.05, **p < 0.007, ****p < 10^−5^. The error bars are derived from triplicates. (**E**) Proposed model. CD56^bright^ CD16^−/dim^ NK cells migrate out of the peripheral blood to the joint of RA patients. In the peripheral blood, CD56^bright^ CD16^−/dim^ NK cells (green) mainly express CXCR3 and CCR1 and migrate towards their ligands present in the SFs of RA patients. In the SFs, the CD56^bright^ CD16^−/dim^ NK cells decrease the expression of CXCR3, acquire the expression of other chemokine receptors (mainly CXCR4, but also CCR5 and CCR7) and become activated. Around half of the sfNK cells in DRA patients differentiate into the CD56^dim^CD16^+^ NK cells. Upon interaction with IL-15 and IL-2 sfNK cells secrete TNFα and IFNγ.

Finally, we investigated the functionality of NK cells isolated from blood and SFs of DRA and NDRA patients. To test this we incubated an equal number of the various NK populations in medium containing rhIL-15, an abundant cytokine in the SFs of RA patients^26^, also known to activate NK cells^27^. As a control, we incubated the same number of blood and sfNK cells with rhIL-2, another cytokine known to potentiate both growth and cytotoxic functions of NK cells^27^. Next, we assessed the levels of TNFα and IFNγ secreted from the NK cells incubated with either IL-2 or IL-15. SfNK cells from DRA patients secreted significantly higher levels of TNFα and IFNγ than all NK cells tested (Fig. 5A–D). Furthermore, sfNK cells from DRA patients secreted relatively more IFNγ in the presence of IL-15 as compared to IL-2 (Fig. 5C,D). To note, no changes of the receptor repertoire expression (shown in Fig. 2) was observed when the pool of NK cells isolated from the blood or SFs of both NDRA and DRA patients were incubated with either IL2 or IL-15 (Fig. S4A).

## Discussion

RA varies among patients, ranging from a mild inflammatory disease with a small impact on patients’ functional capacity to a severe erosive disease accompanied by joints subluxations, deformities, contractures, and subsequent poor quality of life. Bone erosion begins early in the course of RA, constitutes a key outcome measure in RA, and is predictive of a more severe course of disease. The prospect of distinguishing which patient with early RA may develop severe erosive deformative disease and who may not is of great value in clinical practice. There are several potential prognostic factors for joint damage in RA known today, such as demographic, clinical, inflammatory markers, autoantibodies, bone markers and early imaging damage^28^. However, additional prognostic factors which may predict future patient outcomes are desperately needed. Identifying these markers would enable proper treatment of patients who have the potential of developing a severe disease accompanied with irreversible structural damage.

As such, this study focused on a type of immune cell, NK cell, which was previously known to reside within the SFs of RA patients, and to be important in bone destruction and erosion formation both in *in vitro* and *in vivo* models of arthritis^19^.

In our study we characterized the SFs NK cells of RA patients relative to their disease severity. Our study included 51 patients with RA, some of whom have severe deformative/erosive (at least one erosion visible on x-ray film) disease (DRA), whereas others have milder non-deformative disease (NDRA). Most of the patients were on combined DMARDs and biological treatments. The average disease activity score (based on clinical arthritis parameters and CRP, and expressed as DAS28CRP) was 5.1 (high disease activity) in the DRA group, and 4.5 (moderate disease activity) in the NDRA group. In addition, most of the patients were both positive for rheumatoid factor (RF), and anti-cyclic citrullinated peptide (anti CCP) antibodies, which are diagnostic and prognostic factors for RA.

We show here that in healthy individuals and NDRA patients, NK cells comprise around 10% of peripheral blood lymphocytes, but in patients with established active DRA, around 25% of all lymphocytes were NK cells. NK cells were also found within the SFs of both DRA and NDRA patients, but their percentages were significantly higher in the DRA patient group (21% in DRA as opposed to 6% in NDRA patients). An interesting trend was also observed in both RA patient groups, where the percentage of NK cells within the SFs seemed to correlate with disease activity (DAS28CRP). For example, patients 1, 3, 6, 17 in the DRA (Table 1), and patients 6, 16, and 27 in the NDRA group (Table 2), all have very high disease activity scores (DAS28CRP~6.0), and they also presented with the highest percentages of sfNK cells (Fig. S5A,B). On the other hand, several patients with severe deformative-erosive disease but with low disease activity (burned out disease) had low percentages of NK cells (Fig. S5A,B). Thus, while sfNK cells may be a useful disease biomarker, a direct correlation between sfNK total NK cells (and NK cell subsets) and disease activity was not consistently seen throughout our small study cohort (Figs S5A,B, and S6A,B) and should be more thoroughly investigated in future studies. It is also possible that sfNK cell numbers are affected by disease time course and specific pharmacological therapy, which may explain differences observed between the two groups.

Consistent with a previous study^17^, we observed that the distribution of the NK cells subsets within the SFs was drastically different from the distribution typically seen within the blood. Blood NK cells are made up of around 10% CD56^bright^ and 90% CD56^dim^ NK cells, whereas the NK cells in the SFs of DRA and NDRA patients had a highly significant expansion of the CD56^bright^ phenotype (37.7 ± 7.9 in NDRA and 39.9% ± 10.4 in DRA, Fig. 1). The sfNK cells displayed an activated phenotype compared to blood NK cells. We observed high levels of CD69 and an overall increase in NKp46 expression on sfNK cells of both DRA and NDRA patients. CD69 is a C-type lectin receptor shown to be induced following NK cell activation *in-vitro*. It also serves as a tissue residence marker^21^. NKp46 is one of the most potent activating receptors expressed on NK cells^5^, and upregulation of NKp46 indicates that NK cells are activated^22^.

The two sfNK subsets (CD56^dim^ and CD56^bright^) are very similar to each other. One major difference was noted between these two sfNK populations: the CD56^dim^ sfNK cells upregulate the NKp46 receptor, whereas CD56^bright^ sfNK cells slightly downregulate NKp46 relative to the blood NK cells. The reasons for this phenomenon are currently unknown.

The observation that the dominant subset of sfNK cells of DRA and NDRA patients had the CD56^bright^ phenotype suggests that the CD56^bright^ population might migrate from the blood into the SFs (Fig. 5E). When focusing on this subpopulation we found that the expression of CXCR3 is decreased only in the CD56^bright^ sfNK subset compared to the blood. The downregulation of CXCR3 on the sfNK cells may be a consequence of ligand binding and internalization of the chemokine receptor^29^, suggesting that upon migration into the synovium the CD56^bright^ NK cells downregulate CXCR3. Indeed, high levels of CXCR3 ligands such as IP-10, and Mig (monokine induced by interferon-gamma) are present in the SFs of RA patients^30–32^. We further found increased expression of CCR1 on sfNK cells, and relative to the blood (in which CCR1 is expressed mainly on the CD56^bright^ subset), higher expression of the CCR1 on the SF CD56^dim^ subset. These findings suggest preferential migration from the blood of the CD56^bright^ NK cells, and thus may hint that the SF CD56^dim^ subset originated from the blood CD56^bright^ subset. The migration of blood CD56^bright^ NK cells can also explain the low expression of CXCR1 and CX3CR1 on sfNK cells which are mainly expressed on the blood CD56^dim^ NK cells. Expression of CXCR4 is up-regulated on the major subset of sfNK cells, whereas, CCR5 and CCR7 are up-regulated on a minor subset of sfNK cells. These chemokine receptors were shown to be important in homing and retention of immune cells in bone marrow, lymph nodes and in inflamed tissues^33^. In contrast with our results, Dalbeth *et al*.^17^ showed that the majority of the SF NK cells express the chemokine receptors CXCR3 and CCR5. The discrepancies between the studies are unclear, but it seems that the RA patient populations are different (as described above, in our study most of the RA patients were on combined DMARDs and/or biological treatments, above 85% of the patients were seropositive and had moderate to high active disease). Similar to Dalbeth *et al*.^17^, we also observed that most of the SF NK cells did not expressed the KIR receptor.

We suggest that once in the inflamed joint, NK cells undergo changes mediated by the pro-inflammatory surrounding and participate in the pathogenesis of RA disease (Fig. 5E). We further suggest that CD56^bright^ NK cells undergo partial maturation once they reach the SF. Indeed, approximately half of the DRA and NDRA sfNK CD56^bright^ population expressed the low affinity Fc receptor CD16 (Fig. 3A). Such distribution of CD16 on CD56^bright^ NK cells has never been observed before in any other organ^34^. The CD56^bright^ CD16^−^ NK cells are considered to be the precursor cells of the CD56^dim^CD16^+^ subset, and CD56^bright^ CD16^+^ cells are observed during NK cell development and maturation as previously described^9,25^.

Supporting our model, it was previously shown that heterophilic adhesion of CD56^bright^ NK cells with synovial fibroblasts via a CD56-FGFR1 interaction, may aid the differentiation into CD56^dim^CD16^+^ NK cells^35^.

Following phenotype characterization of blood and sfNK cells in two groups of RA patients, we proceeded to explore whether their functions differ. We isolated sfNKs from DRA and NDRA patients and incubated them with either IL-2 or IL-15. Interestingly, the sfNKs from DRA patients secreted the highest levels of TNFα and IFNγ when incubated with IL-15. IL-15 is a cytokine present in the SFs of RA patients^26^, previously shown to be important in disease progression in both human and mice, and its amount in the SF correlates strongly with disease severity in patients with established disease^36,37^.

IL-15 is also required for the development, survival, and activation of NK cells^27,38^, and is important for the development of cytokine-induced memory-like NK cells^14,15,39^. It was shown that human NK cells exposed to IL-12/IL-15/IL-18, washed and then cultured in IL-15 for additional days produce elevated amounts of IFNγ upon re-stimulation with IL-15^38^. The sfNK cells are exposed to abundant amounts of IL-12/IL-15/IL-18 in the SFs of patients with RA^26^. We therefore suggest that the more severe inflammatory environment present in the joints of DRA patients in comparison to NDRA patients may enhance response of sfNK cells to IL-15 similar to the cytokine-induced memory-like phenotype previously reported^14,15,39^.

In summary, our study has revealed that patients with advanced RA have higher percentages of activated sfNK cells compared with patients with milder non-deformative disease. We thus propose that sfNK cells levels may be a marker for RA disease severity and their presence in high numbers could serve as a red flag for the instigation of earlier and more aggressive monitoring and therapy.

## Methods

### Ethics Statement and patients description

The institutional Helsinki committee of Hadassah approved the study (Helsinki number 0030-12-HMO and Helsinki number 0353-15-HMO). All subjects provided informed consent.

Peripheral blood was obtained from 24 patients with DRA (Table 1) and 27 patients with NDRA (Table 2). SFs were obtained from 16 patients with DRA and 18 patients with NDRA, who presented to the clinic with knee effusions (overall, 16 and 17 paired samples of blood and SFs were taken, respectively). All patients were classified based on the 2010 American College of Rheumatology/European League against Rheumatism criteria^40^. Deformative-Erosive RA was defined in patients with established RA by having deformations prototypical for RA (e.g. ulnar deviation, swan-neck and boutonniere), and at least one erosion visible in x-ray films. The average age was 64.5 ± 11.5 in the DRA group and 57.96 ± 13.97 in the NDRA group (p = 0.075). The DRA group included 41.7% males and 58.3% females, whereas the NDRA group included 29.6% males and 70.4% females (p = 0.396). The mean disease duration was 15.9 + 9.1 in the DRA group and 11.22 + 6.58 in the NDRA group (p = 0.0395). 91.7% versus 85.2% of DRA and NDRA patients, respectively were positive for either RF or anti-CCP antibodies (p = 0.067). In both patient groups, about 63% of the patients were treated with biological treatments (p = 1.00). The average disease activity of the patients measured by the DAS28CRP score (Disease Activity Score in 28 joints using the C-reactive protein level) describes severity of rheumatoid arthritis using clinical scoring (considering count for tenderness and swollenness of 28 joints and patient global assessment) and laboratory inflammatory index of C-reactive protein level, indicated in Tables 1 and 2 and in Figs S5 and S6. The average DAS28CRP in the DRA group was 5.1 ± 1.178 and 4.48 ± 1.159 in the NDRA group (p = 0.114). As controls, we used samples of peripheral blood obtained from 32 healthy individuals (Table 3, the control group included 31.2% males and 68.8% females, the average age was 56.8 ± 12.9).

### Isolation of mononuclear cells from blood and synovial fluid

Heparinized blood was loaded on Lymphoprep^TM^ (StemCells Technologies) gradient and PBMCs were purified. Synovial fluids from DRA patients were filtered through 70 µm PET strainers (Greiner bio-one) and then loaded on a Lymphoprep^TM^ gradient. PBMCs and synovial fluid cells were stained with anti-CD56-Phycoerythrin, anti-CD16-FITC and anti-CD3-Allophycocyanin (all from Biolegend) to distinguish between subpopulations.

The blood and SF PBLs samples used in the experiments were kept frozen in −80 °C.

### FACS staining and antibodies

FACS staining was performed using standard procedures. Staining was performed with the following conjugated antibodies: anti CD56, anti CD3, anti CD16 (all from Biolegend), anti 2B4, anti NKp46, anti CD69 (BD), anti NKp30, anti NKp44, anti NKG2D, anti DNAM1, anti NKG2C (R&D), anti CEACAM1 (R&D), anti TIGIT (eBioscience), anti NKG2A (R&D). Antibodies against chemokine receptors that were used included anti CCR1, anti CCR2, anti CCR3, anti CCR5, anti CCR7, anti CCR10, anti CXCR1, anti CXCR2, anti CXCR3, anti CXCR4, and anti CX3CR1. Other antibodies used were: anti CD57 and antibodies against the killer cell immunoglobulin-like receptors-KIR2DL1/DS1, anti KIR2DL2/DL3, and anti KIR3DL1/KIR3DS1. Unless otherwise noted, all antibodies in this study were purchased from Biolegend. In all FACS results shown in this paper, dead cells, neutrophils, and monocytes were excluded from analysis. We used the FCS Express version 4 program for FACS analysis. We performed correction for the MFI of different unrelated FACS staining, by dividing the individual staining MFI (of blood and SFs NK cells) by the background isotype control of the same staining (normalized MFI).

### Cytokine secretion from synovial fluids NK cells

PBMCs were purified from heparinized blood by centrifugation on Lymphoprep (StemCells Technologies). NK cells were isolated using the EasySep human NK cell enrichment kit (StemCells Technologies). The generation and culturing of activated NK cells were described previously^29^. In short, activated NK lines were generated by culturing isolated NK cells together with irradiated feeder cells (allogeneic PBMCs from two donors and RPMI-8866 cells in each well) and 20 mg/ml PHA (Roche). Both PBMCs and RPMI-8866 cells were irradiated in 6,000 rad prior to seeding in 96-well U-bottom plate. The cultures were maintained in DMEM:F-12 Nutrient Mix (70:30), 10% human serum (Sigma-Aldrich), 2 mM glutamine (Biological Industries [BI]), 1 mM sodium pyruvate (BI), 13 nonessential amino acids (BI), 100 U/ml penicillin (BI), 0.1 mg/ml streptomycin (BI), in the presence or absence of 0.1 µg/ml rhIL-15 (BLG-570302) or 500 U/ml rhIL-2 (Peprotech, 200-02-1000) at 37 °C and 5% CO2. Blood NK cells pooled from 6 different healthy control, and 9 and 10 different DRA and NDRA patients, respectively. Synovial NK cells were pooled from matched 9 and 10 different patients with DRA and NDRA, respectively. The purified population was stained with antibodies against CD56 and CD3 as well as corresponding isotype controls. The purity of the blood and synovial NK cells was checked and found to be higher than 94% (Fig. S4B).

For the cytokine secretion experiments we incubated 5 × 10^4^ NK cells/well derived from blood and SFs of control, DRA, and NDRA patients. After 48 hours we counted the number of live cells (using FACS, with time run of 30 seconds) present in the different conditions. Levels of the cytokines IFNγ and TNFα in supernatants were measured by ELISA using matching antibodies against IFNγ (pair BLG-502402, 502504), and TNFα (pair BLG-508402, 508502).

### Statistical analysis

Data are presented as means ± standard deviations (SD) or standard errors (SE). Two-tail *t*-tests were used to compare between groups. For more than two groups, One-Way ANOVA as implemented by SPSS (IBM SPSS Statistics for Windows, Version 23.0, 2015. Armonk, NY: IBM Corp.) was employed, followed by an all pairwise comparisons test (Dunnett’s *post-hoc* test was chosen to correct for the multiple comparisons because it does not require homogeneity of variances and a Levene test was highly significant). Fisher’s Exact Test was used for all 2 × 2 tables.

Statistical significance was uniformly set at a maximum *p*-value of 0.05.

All experiments and experimental protocols described in this paper were performed in accordance with the Hebrew University safety and ethics guidelines and regulations.

## Supplementary information

Supplementary figures
