# Supplementary material for: High percentages and activity of synovial fluid NK cells present in patients with advanced stage active Rheumatoid Arthritis
Source: Sci Rep. 2019 Feb 4;9:1351. doi: 10.1038/s41598-018-37448-z (PMC6361912; doi:10.1038/s41598-018-37448-z)
Supplement: Supplementary file 1 — Supplementary figures [file 41598_2018_37448_MOESM1_ESM.pdf]

High percentages and activity of synovial fluid NK cells present in patients with advanced stage active Rheumatoid Arthritis

Rachel Yamin , Orit Berhani, Hagit Peleg, Suhail Aamar, Natan Stein, Moriya Gamliel, Issam Hindi , Anat Scheiman-Elazary, and Chamutal Gur

Fig. S1

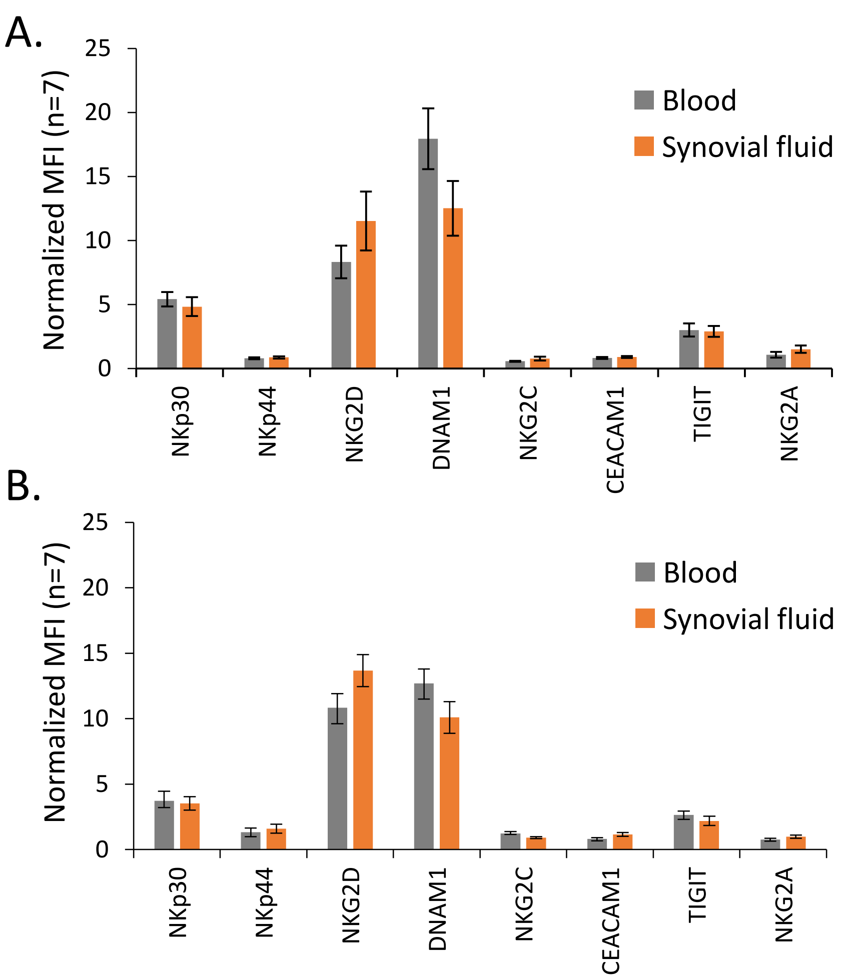

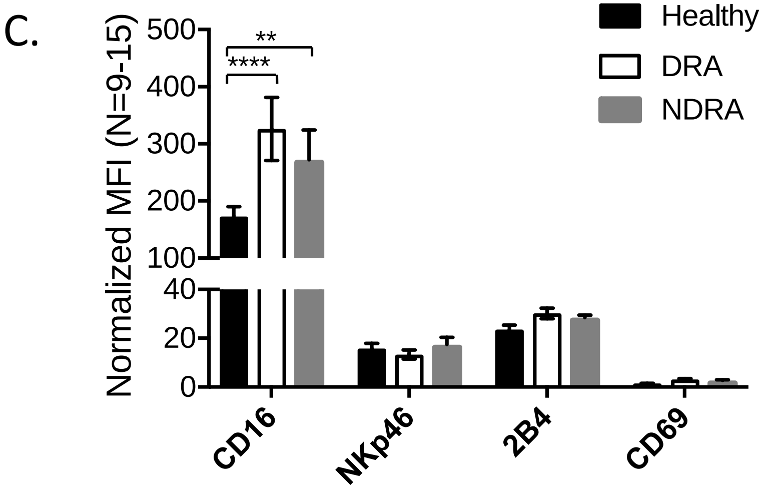

Figure S1. Alterations in NK receptors expressed on blood and SF NK cells of RA patients- related to figures 2.

(A-B) Mononuclear cells from peripheral blood (gray bars) or synovial fluid (orange bars) of DRA patients (A) or NDRA patients (B) were triple stained with anti CD56, anti CD3, and various monoclonal antibodies directed against different NK receptors (indicated on X-axis). Figure shows summary data of FACS stainings from 7 donors. Data is presented as mean ± SE (n=7). (C) Mononuclear cells from peripheral blood of healthy control, DRA, and NDRA patients (n=9-15) were triple stained with anti CD56, anti CD3, and various monoclonal antibodies directed against different NK receptors. Summary data of FACS stainings for the CD16, 2B4, NKp46 receptors and CD69 are presented as mean ± SE. **p<0.002, ****p<0.0001.

Fig. S2

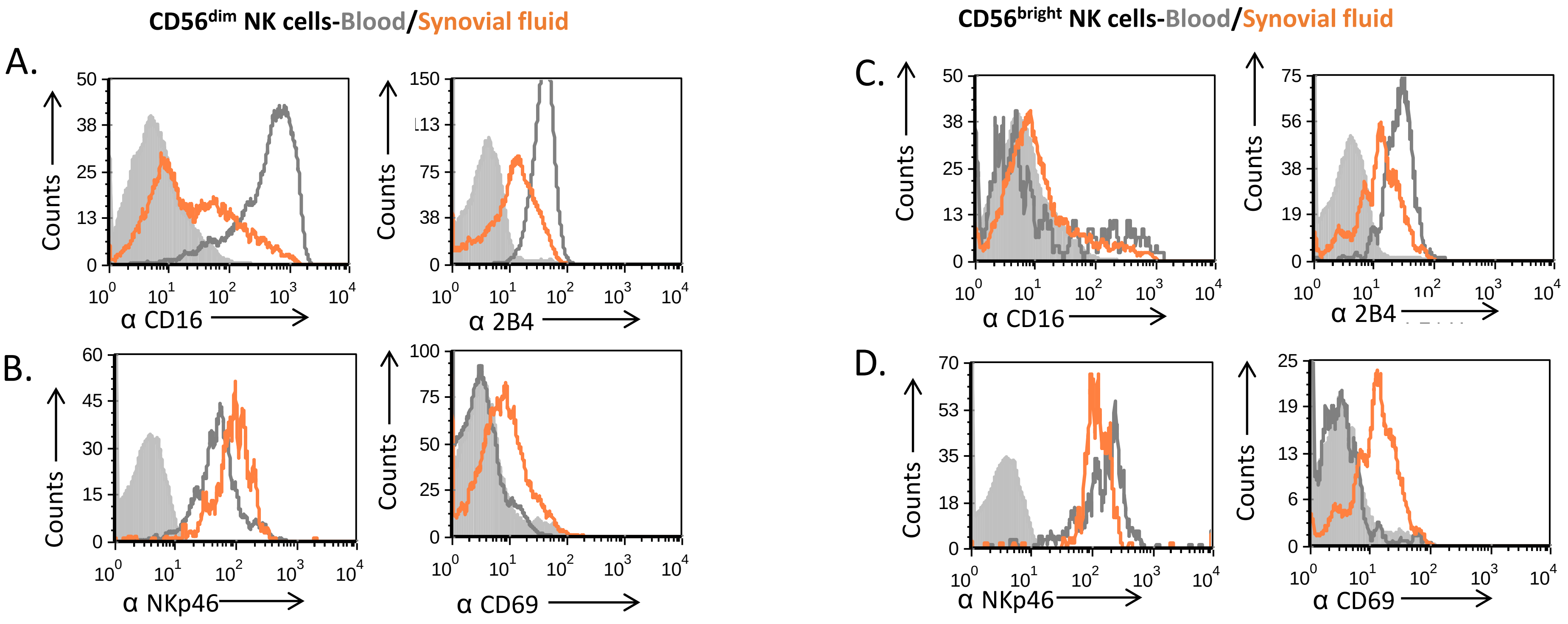

Figure S2. Synovial NK cells are a unique population

(A-D) Mononuclear cells from peripheral blood or SFs of NDRA patients were quadruple stained with anti CD56, anti CD3, and various monoclonal antibodies directed against the different NK receptors. CD56 ^{dim} NK cells and CD56 $^{+ bright}$ NK cells were gated, and the expression of NK receptors CD16 (A and C, left), 2B4 (A and C, right), NKp46 (B and D, left), and CD69 (B and D, right) was determined. Figure shows staining of cells from one representative donor out of 6 described in Figure 2 that were tested. Open gray histograms show the staining of the receptors on cells from the peripheral blood. Open orange histograms indicate cells from the SFs. Filled gray histograms show the background staining of cells from the SFs with an isotype control. The backgrounds of the PBMCs were similar to the SFs cells and are not shown in the figure.

Fig. S3

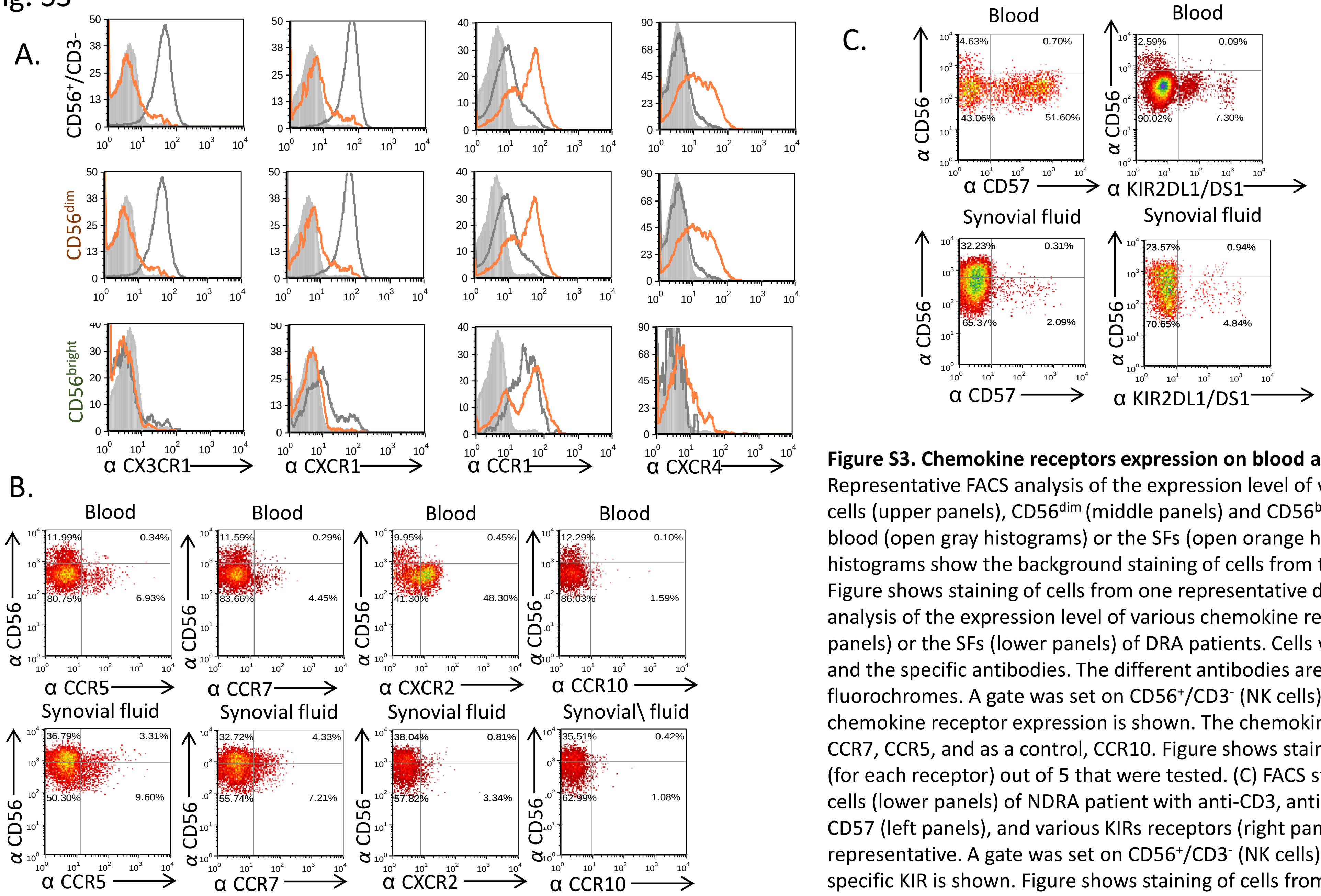

Figure S3. Chemokine receptors expression on blood and SFs of DRA and NDRA patients (A) Representative FACS analysis of the expression level of various chemokine receptors on whole NK cells (upper panels), CD56^{dim} (middle panels) and CD56^{bright} (lower panels) NK subsets from the blood (open gray histograms) or the SFs (open orange histograms) of NDRA patient. Filled gray histograms show the background staining of cells from the SFs with an isotype control. Figure shows staining of cells from one representative donor out of 6 that were tested. (B) FACS analysis of the expression level of various chemokine receptors on NK cells from the blood (upper panels) or the SFs (lower panels) of DRA patients. Cells were stained with anti-CD3, anti-CD56, and the specific antibodies. The different antibodies are conjugated to three different fluorochromes. A gate was set on CD56^{+}/CD3^{-} (NK cells), and CD56 expression versus specific chemokine receptor expression is shown. The chemokine receptors that are shown are: CXCR2, CCR7, CCR5, and as a control, CCR10. Figure shows staining of cells from one representative donor (for each receptor) out of 5 that were tested. (C) FACS staining of blood (upper panels) and SFs NK cells (lower panels) of NDRA patient with anti-CD3, anti-CD56, and specific antibodies against CD57 (left panels), and various KIRs receptors (right panels), with KIR2DL1/DS1 shown as a representative. A gate was set on CD56^{+}/CD3^{-} (NK cells), and CD56 expression versus CD57 or specific KIR is shown. Figure shows staining of cells from one representative donor (for each receptor) out of 5 that were tested.

Fig. S4

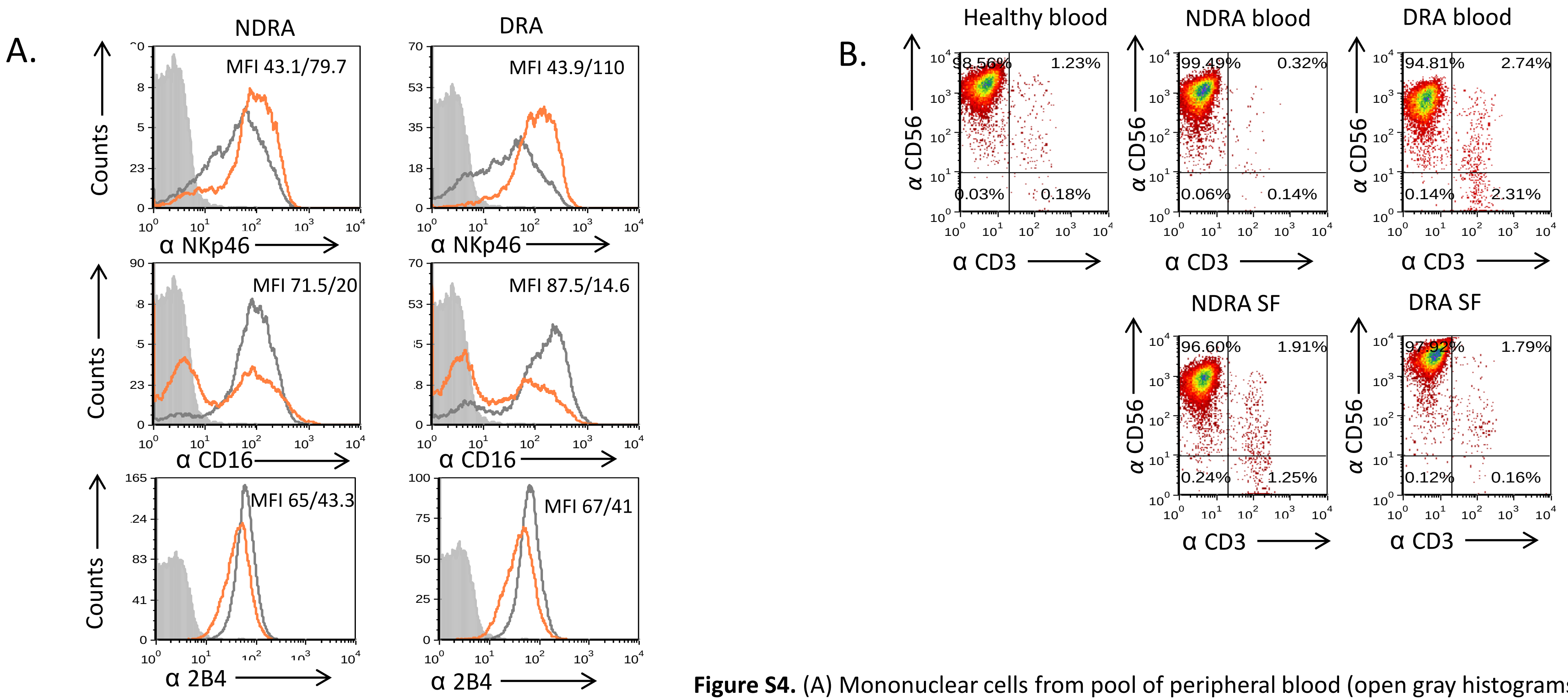

Figure S4. (A) Mononuclear cells from pool of peripheral blood (open gray histograms) or SFs (open pink histograms) of 9 DRA (right panels), and 10 NDRA (left panels) patients described in Figure 5A-D, were triple stained with anti CD56, anti CD3, and various monoclonal antibodies directed against NKp46 (upper), CD16 (middle), and 2B4 (lower) NK receptors. Filled gray histograms show the background staining of cells from the SFs with an isotype control. Figure shows one staining of cells derived from pool of patients out of 4 were performed.
(B) Purity of NK cells ($CD56^+/CD3^-$) sorted from a pool of blood of 6 normal controls and a pool of blood and SFs of 9 DRA and 10 NDRA patients described in table 1-3.
.

Fig. S5

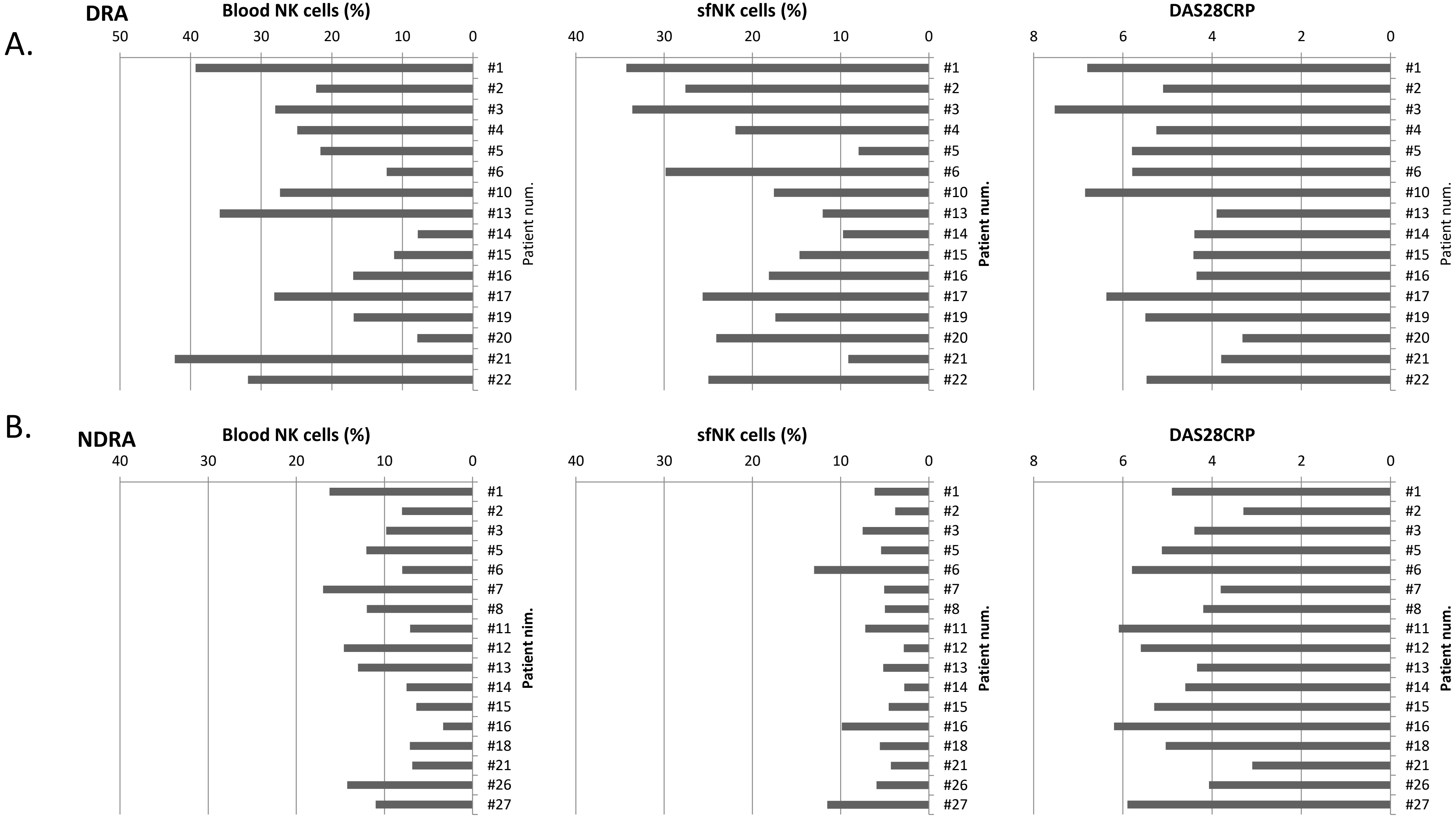

Figure S5. Plots of paired samples of blood and sfNK cells derived from DRA (A) and NDRA (B) patients presented in Table 1-2, gated vs DAS28CRP.

Fig. S6

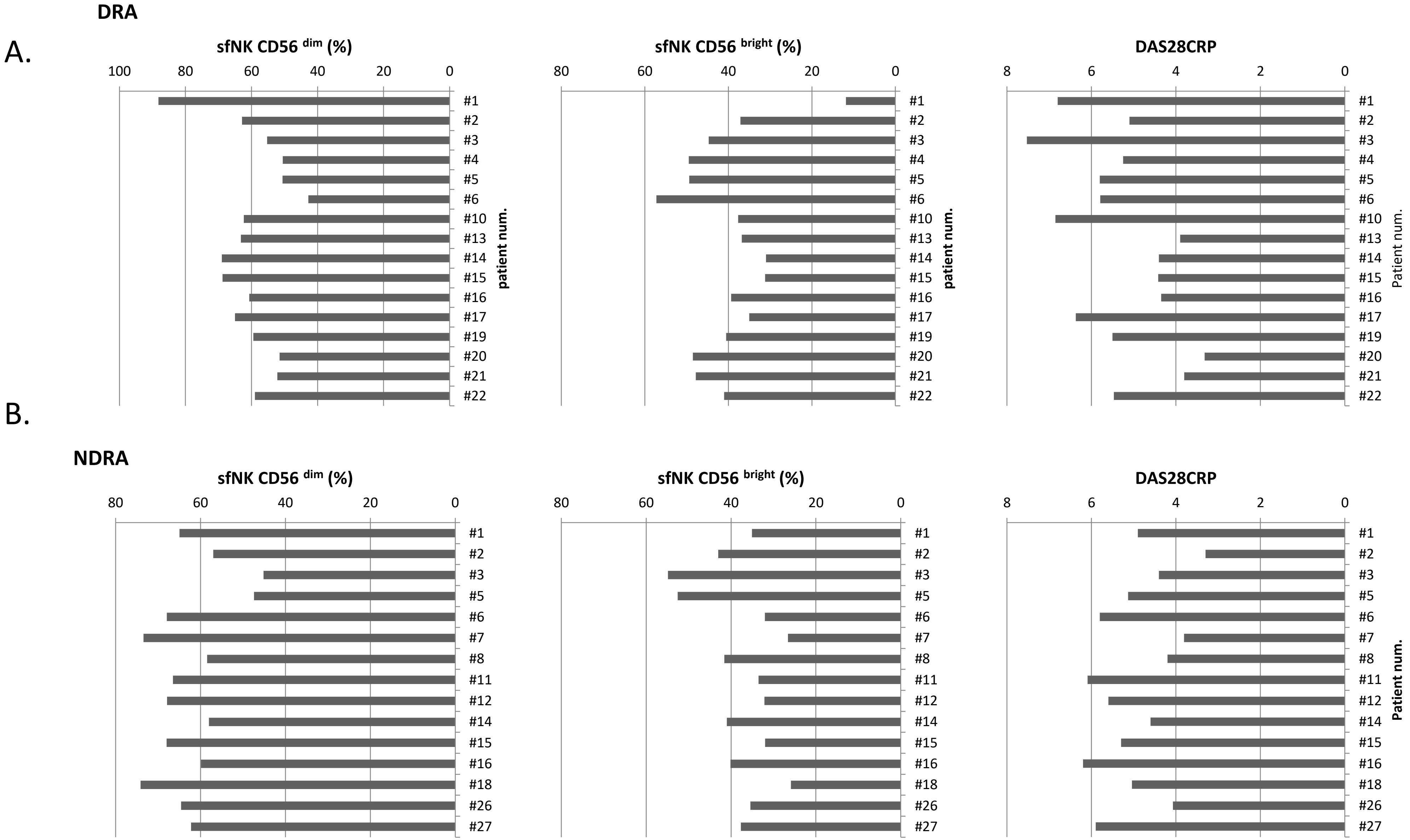

Figure S6. Plots of paired samples of blood and sfNK CD56dim and CD56 bright cells derived from DRA (A) and NDRA (B) patients presented in Table 1-2, gated vs DAS28CRP.
